# Supplementary material for: Research on compound twill generation algorithm based on moving matrix
Source: PLoS One. 2025 May 9;20(5):e0322531. doi: 10.1371/journal.pone.0322531 (PMC12063832; doi:10.1371/journal.pone.0322531)

# **Figure Information**

**S1 Fig.**


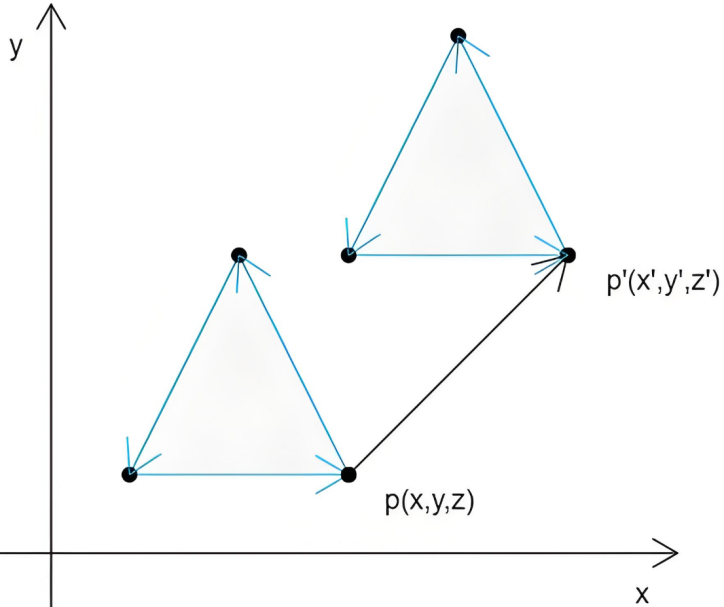


**S2 Fig.**


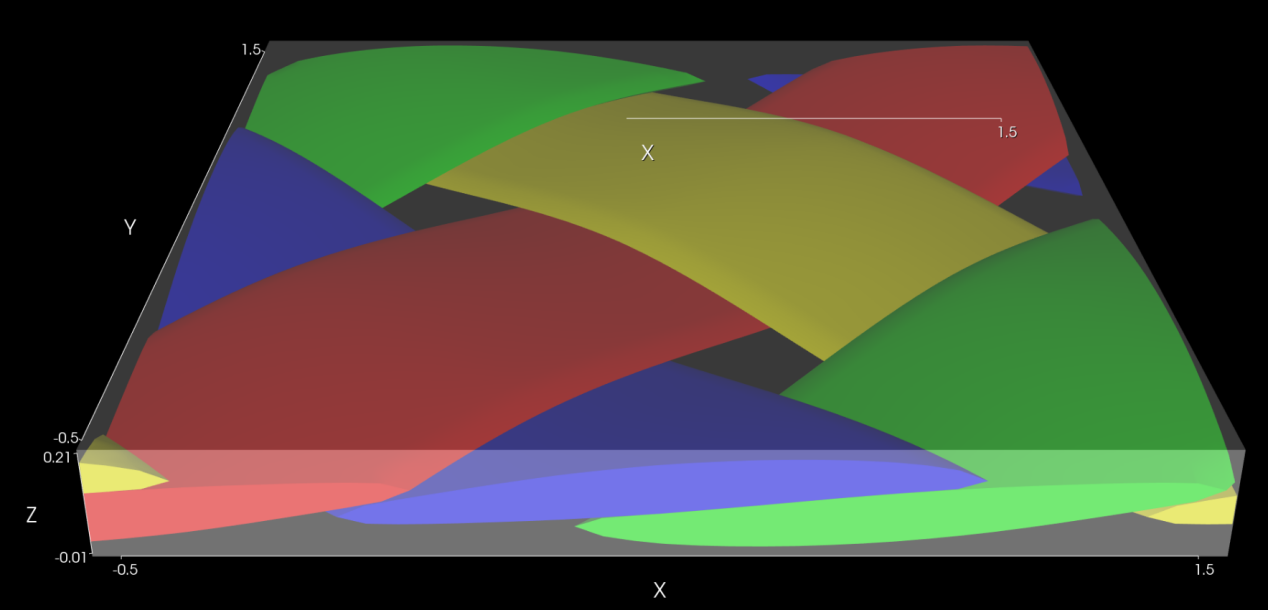


**S3 Fig.**


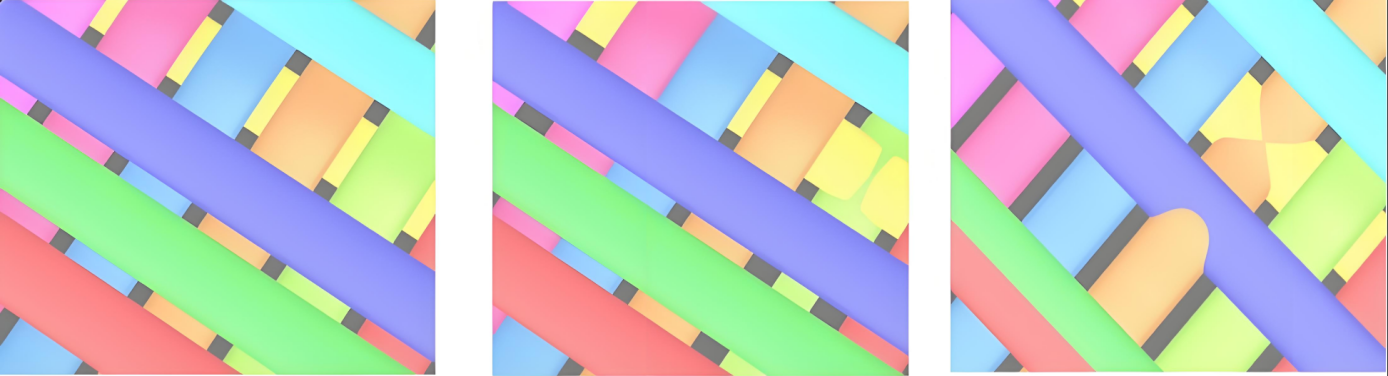


(a) Effect diagram of moving matrix method

(b) Effect diagram of Hilbert algorithm

(c) Effect diagram of Bessel algorithm

**S4 Fig.**


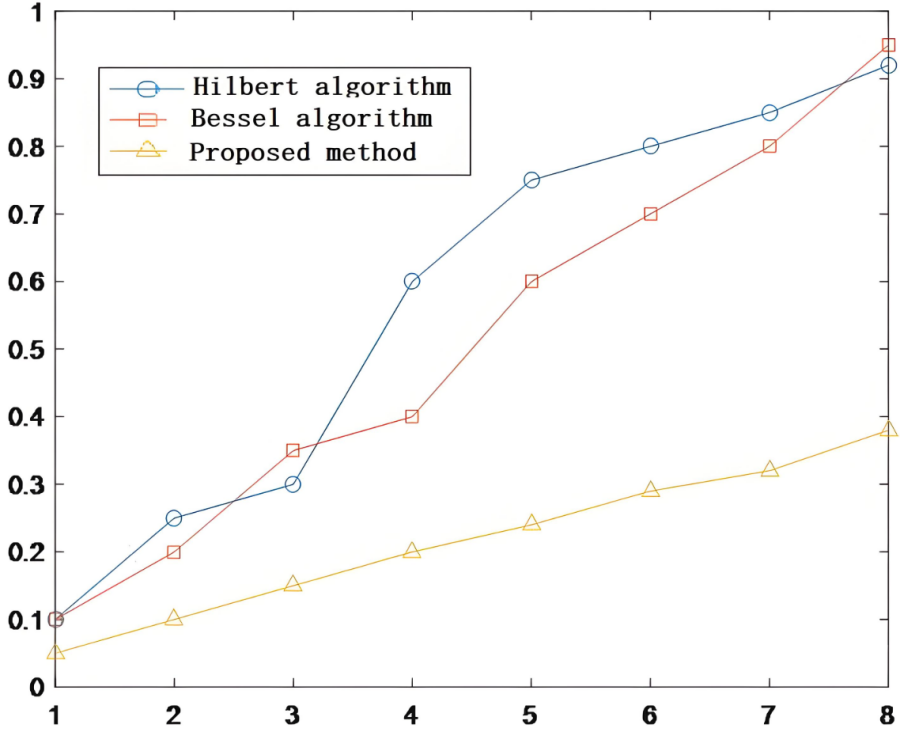


**Number of test steps**

**Running time (unit:s)**

**S5 Fig.**


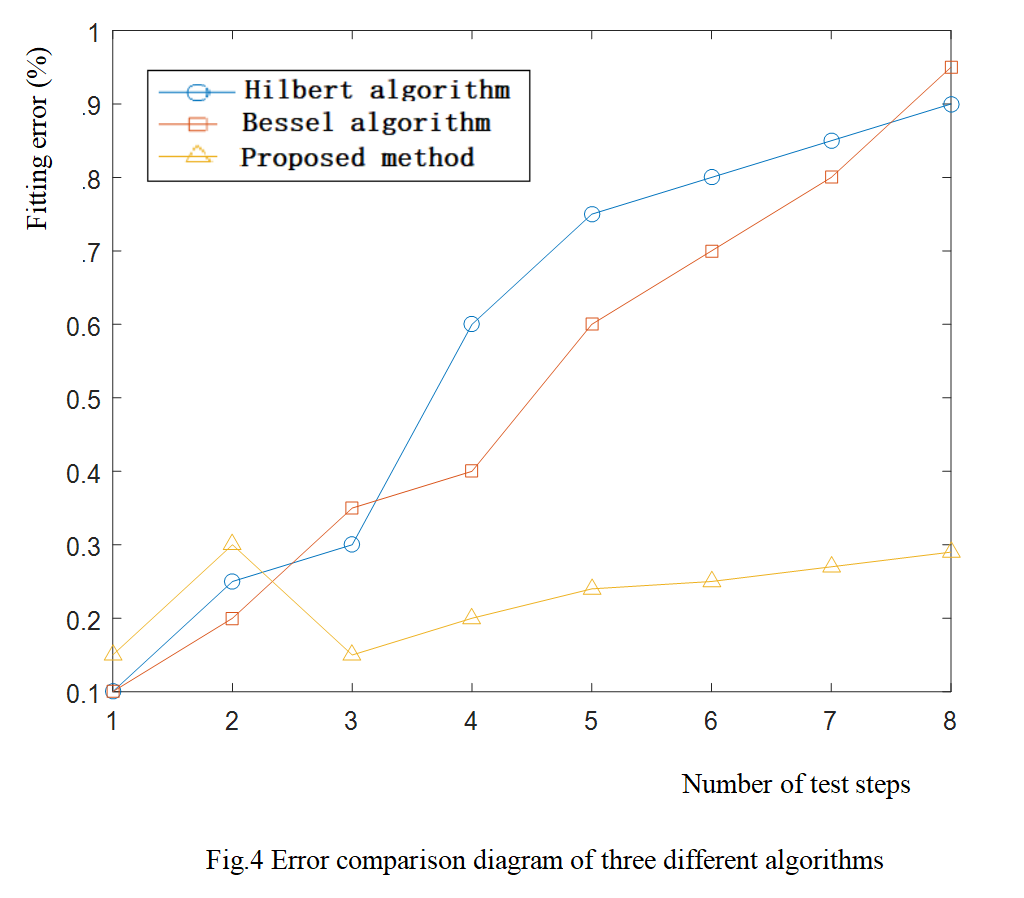

Supplement: S1-S5 Figs — (DOCX) [file pone.0322531.s001.docx]
