# Supplementary material for: Research on compound twill generation algorithm based on moving matrix
Source: PLoS One. 2025 May 9;20(5):e0322531. doi: 10.1371/journal.pone.0322531 (PMC12063832; doi:10.1371/journal.pone.0322531)
Supplement: S1 File — (DOCX) [file pone.0322531.s002.docx]

Supporting Information

**Comparison data of errors of three different algorithms**

| algorithm |  | | | | | | | |
| --- | --- | --- | --- | --- | --- | --- | --- | --- |
| Hilbert | Test step number | | | | | | | |
|  | 1 | 2 | 3 | 4 | 5 | 6 | 7 | 8 |
|  | Test error (%) | | | | | | | |
|  | 0.1 | 0.25 | 0.3 | 0.6 | 0.75 | 0.8 | 0.85 | 0.90 |
| bezier | Test step number | | | | | | | |
|  | 1 | 2 | 3 | 4 | 5 | 6 | 7 | 8 |
|  | Test error (%) | | | | | | | |
|  | 0.1 | 0.2 | 0.35 | 0.4 | 0.6 | 0.7 | 0.8 | 0.95 |
| This article's method | Test step number | | | | | | | |
|  | 1 | 2 | 3 | 4 | 5 | 6 | 7 | 8 |
|  | Test error (%) | | | | | | | |
|  | 0.15 | 0.3 | 0.15 | 0.2 | 0.24 | 0.25 | 0.27 | 0.29 |

**Comparison data of running time of 3 algorithms**

| algorithm |  | | | | | | | |
| --- | --- | --- | --- | --- | --- | --- | --- | --- |
| Hilbert | Test step number | | | | | | | |
|  | 1 | 2 | 3 | 4 | 5 | 6 | 7 | 8 |
|  | Running time (unit:s) | | | | | | | |
|  | 0.1 | 0.24 | 0.29 | 0.6 | 0.75 | 0.8 | 0.83 | 0.9 |
| bezier | Test step number | | | | | | | |
|  | 1 | 2 | 3 | 4 | 5 | 6 | 7 | 8 |
|  | Running time (unit:s) | | | | | | | |
|  | 0.1 | 0.2 | 0.35 | 0.39 | 0.58 | 0.67 | 0.78 | 0.92 |
| This article's method | Test step number | | | | | | | |
|  | 1 | 2 | 3 | 4 | 5 | 6 | 7 | 8 |
|  | Running time (unit:s) | | | | | | | |
|  | 0.05 | 0.09 | 0.13 | 0.18 | 0.22 | 0.26 | 0.31 | 0.35 |
