# Supplementary material for: Research on compound twill generation algorithm based on moving matrix
Source: PLoS One. 2025 May 9;20(5):e0322531. doi: 10.1371/journal.pone.0322531 (PMC12063832; doi:10.1371/journal.pone.0322531)
Supplement: S1-S3 Tables — (DOCX) [file pone.0322531.s003.docx]

# **Table Information**

**S1 Table**

| Algorithm type | Mean fitting error (%) | Standard deviation (%) |
| --- | --- | --- |
| Hilbert algorithm | 0.5678 | 0.3093 |
| Bessel algorithm | 0.5125 | 0.2985 |
| Mobile matrix method | 0.2313 | 0.0589 |

**S2 Table**

| Algorithm type | Mean fitting error (%) | Standard deviation (%) |
| --- | --- | --- |
| Hilbert algorithm | 0.6138 | 0.3712 |
| Bessel algorithm | 0.4532 | 0.2536 |
| Mobile matrix method | 0.2035 | 0.0758 |

**S3 Table**

| Algorithm type | Mean fitting error (%) | Standard deviation (%) |
| --- | --- | --- |
| Hilbert algorithm | 0.6375 | 0.3816 |
| Bessel algorithm | 0.4126 | 0.2413 |
| Mobile matrix method | 0.2105 | 0.0875 |
