# Supplementary material for: Research on compound twill generation algorithm based on moving matrix
Source: PLoS One. 2025 May 9;20(5):e0322531. doi: 10.1371/journal.pone.0322531 (PMC12063832; doi:10.1371/journal.pone.0322531)
Supplement: Attachment — Submitted filename: Response to Reviewers-PLOS ONE.docx [file pone.0322531.s005.docx]

Response to Reviewers

According to the reviewer's opinion, the response is as follows.

Reviewer #1

1. The abstract could benefit from more clarity. For example, explain "changing flying number of twill curves" more explicitly to improve accessibility for readers less familiar with the terminology.

Reply: The key to designing composite twill is to determine the fly count. When the fly count of the twill is constantly changing, a composite twill can be obtained. Therefore, by utilizing the constantly changing characteristics of the fly count of the composite twill, a composite twill movement matrix model is established. The abstract section has been improved with blue text.

2. Include specific numerical results (e.g., fitting accuracy values) in the abstract to provide a concise summary of the contributions.

Reply: The abstract supplements the average fitting accuracy of three different algorithms, as shown in the blue text in the abstract section.

3. The introduction discusses previous work effectively but should better emphasize the limitations of current methods. This would help underline the novelty and significance of the proposed algorithm.

Reply: The limitations of the current method are further emphasized, as detailed in the blue text in the introduction.

4. The mathematical derivations are comprehensive, but the explanation of key terms (e.g., "twill flight number") and their physical significance could be expanded to improve understanding.

Reply: More explanations have been added for key terms (such as "twill flight number" and " number of organizational cycles") and their physical meanings, as well as the blue text in the section on the moving matrix model.

5. Adding more visual aids (e.g., diagrams illustrating the moving matrix concept) would help clarify the methodology.

Reply:Added a description of the concept of the moving matrix model, as shown in Figure 1.

6. While the comparison with Hilbert and Bessel algorithms is compelling, more details on the experimental setup (e.g., test parameters and conditions) would enhance reproducibility.

Reply:The experimental section has added relevant parameter descriptions, please refer to the blue text in the comparative experimental section for details.

7. Consider including a table summarizing the performance metrics for different algorithms to complement the figures.

Reply: A table has been added to compare the average fitting errors of three different algorithms, as shown in Table 1.

8. The quality of the figures (e.g., Figure 2 and 3) is suboptimal. Higher-resolution images and better labeling would improve the reader's ability to interpret the results.

Reply: Further improve the resolution of Figures 2 and 3, as detailed in the main text.

9. The discussion of results could be expanded to address potential limitations or constraints of the proposed algorithm (e.g., scalability for different fabric types).

Reply: The conclusion section presents the potential limitations of the algorithm and further improvement ideas, as detailed in the blue text in the conclusion section.

10. Provide more insights into the practical implications of reduced error and increased efficiency for real-world applications.

Reply: The second part of the conclusion supplements methods to improve the efficiency of practical application.

11. The conclusion restates the findings well but could include a brief mention of future research directions, such as extending the algorithm to other textile structures or incorporating additional physical properties.

Reply: The third part of the conclusion briefly supplements future research directions.
