# Supplementary material for: Research on compound twill generation algorithm based on moving matrix
Source: PLoS One. 2025 May 9;20(5):e0322531. doi: 10.1371/journal.pone.0322531 (PMC12063832; doi:10.1371/journal.pone.0322531)
Supplement: Attachment — Submitted filename: Response to Reviewers-PLOS ONE - v2.docx [file pone.0322531.s006.docx]

Response to Reviewers

According to the reviewer's opinion, the response is as follows.

Reviewer #1

1. If the authors have adequately addressed your comments raised in a previous round of review and you feel that this manuscript is now acceptable for publication, you may indicate that here to bypass the “Comments to the Author” section, enter your conflict of interest statement in the “Confidential to Editor” section, and submit your "Accept" recommendation.

Reply: The author believe that they have adequately addressed the concerns raised in the previous round of review and consider the manuscript now ready for publication.

2. Is the manuscript technically sound, and do the data support the conclusions?
   The manuscript must describe a technically sound piece of scientific research with data that supports the conclusions. Experiments must have been conducted rigorously, with appropriate controls, replication, and sample sizes. The conclusions must be drawn appropriately based on the data presented.

Reply: As the research method proposed in this manuscript is a simulation study, comparative analysis data of different simulation algorithms have been provided in the previous review, and these data can support the conclusions obtained.

3. Has the statistical analysis been performed appropriately and rigorously?

Reply: Statistical analysis can be conducted from two aspects: computational efficiency and fitting accuracy, which can comprehensively analyze the reliability of the algorithm proposed in this article.

4. Have the authors made all data underlying the findings in their manuscript fully available?
   The [PLOS Data policy](http://track.editorialmanager.com/CL0/http://www.plosone.org/static/policies.action%23sharing/1/010f01958747e223-0933962d-adb4-44f3-a7e2-2bfc4cb0522a-000000/8rtDVo8rfXuUlZP1bL7_gtKLgfVF2896f9S8fjp6FAY=201) requires authors to make all data underlying the findings described in their manuscript fully available without restriction, with rare exception (please refer to the Data Availability Statement in the manuscript PDF file). The data should be provided as part of the manuscript or its supporting information, or deposited to a public repository. For example, in addition to summary statistics, the data points behind means, medians and variance measures should be available. If there are restrictions on publicly sharing data—e.g. participant privacy or use of data from a third party—those must be specified.

Reply: All the data on which the discovery is based has been provided in the manuscript.

5. Is the manuscript presented in an intelligible fashion and written in standard English?

Reply: The manuscript is presented in an easily understandable manner and written in standard English.

6. Review Comments to the Author

Reply: The author has provided detailed and accurate answers to the above questions.

Reviewer #2

1- The paper compares the proposed moving matrix algorithm with Hilbert curve and Bézier curve generation algorithms, but no justification is provided as to why these algorithms were selected. Are they the most relevant benchmarks for evaluating twill generation? The selection process should be explained based on mathematical or computational similarities.

Reply: The Hilbert curve and Bézier curve generation algorithms are mainstream curve simulation generation algorithms that have been used for texture simulation and emulation of fabrics. By comparing the proposed moving matrix algorithm in this paper with these two algorithms, the reliability of the algorithm proposed in this paper can be highlighted.

2- The experimental results are derived from a single set of fabric parameters. It is unclear whether the results generalize to other fabric types. Additional tests on various twill patterns and fabric densities are necessary to validate robustness.

Reply: Simulation comparison experiments for double-twill and triple-twill fabrics have been added. The comparison results are detailed in the blue text of Tables 2 and 3.

3- The error reduction claim (i.e., 33% lower error compared to other methods) is based on an average error value. However, no standard deviation, confidence intervals, or statistical tests (such as ANOVA or t-tests) are provided to confirm the significance of these differences.

Reply: Standard deviation has been added to confirm the significance of the difference, as detailed in the blue text in Table 1.

4- The iterative process of twill matrix transformations is not analyzed in terms of convergence. Does the matrix reach a steady-state representation over iterations, or does it exhibit chaotic behavior for certain twill types?

Reply: When the test step size reaches 8, the iterative process of the matrix tends to stabilize. After testing, it is also applicable to other diagonal types, as explained in blue text in the original text.

5- The paper repeatedly refers to the "twill flight number" but does not provide a clear definition or mathematical formulation. A more precise explanation, preferably with reference to textile engineering standards, is needed.

Reply: The twill flight number refers to the number of yarns in a twill fabric that are spaced between the corresponding tissue points on two adjacent yarns in the same system. The text has been described in blue text.

6- The study is entirely computational but does not attempt to validate the algorithm using real-world woven fabric structures. Were any physical textile samples tested against the predicted patterns to verify the accuracy of the generated twill structures?

Reply: Due to the simulation nature of this study, if real fabrics are used, they are easily affected by light and color, ultimately affecting the accuracy of the simulation.

7- The input structure for the moving matrix algorithm is not explicitly outlined. Does it require raw fabric images, predefined weave structures, or numerical parameters? A detailed data preprocessing section should be added.

Reply: Added input structure and data preprocessing description for the moving matrix algorithm, please refer to the blue text in the moving matrix model section for details.

8- The movement function defined in Equation (2) follows a simple translation transformation. However, why was this specific movement function chosen? Could alternative movement rules improve the algorithm’s flexibility?

Reply: The movement matrix model defined in equation (2) follows the variation law of twill flight number, so this movement matrix model is chosen.

9- The generated twill structures are presented as idealized models, but real-world fabric production involves factors like thread tension, material elasticity, and weave imperfections. How does the algorithm handle these practical constraints?

Reply: Fabric is a flexible material, and the model can incorporate practical constraints such as line tension and material elasticity to simulate real fabrics.

10- The method is optimized for compound twill weaves, but can it be extended to other complex patterns such as Jacquard, Satin, or Herringbone weaves? Some discussion on algorithm adaptability would be beneficial.

Reply: The moving matrix algorithm has high flexibility and scalability, and can adapt to the generation and optimization of complex patterns such as jacquard, satin, and herringbone by adjusting rules, matrix representations, and optimization objectives.

11- The paper does not mention any failure scenarios where the proposed method might not work effectively. Are there any boundary conditions where the algorithm’s performance deteriorates?

Reply: The mobile matrix algorithm may encounter boundary problems such as large matrix size and high pattern complexity, which can be solved by optimizing algorithm design, introducing techniques such as block computing and sparse matrix representation.

12- The proposed matrix-based approach might require significant memory resources for large-scale textile simulations. A discussion on memory efficiency and potential optimizations should be included.

Reply: The combination of sparse matrix and block computing can effectively solve the memory and performance problems of the moving matrix algorithm in large-scale textile simulation. The blue text at the end of the text provides additional explanation.

7. PLOS authors have the option to publish the peer review history of their article ([what does this mean?](https://track.editorialmanager.com/CL0/https://journals.plos.org/plosone/s/editorial-and-peer-review-process%23loc-peer-review-history/1/010f01958747e223-0933962d-adb4-44f3-a7e2-2bfc4cb0522a-000000/KvAld04iXOJtfirSUPAj0s28je0KxWMgYg8hOQiDi-Y=201)). If published, this will include your full peer review and any attached files.
If you choose “no”, your identity will remain anonymous but your review may still be made public.
Do you want your identity to be public for this peer review? For information about this choice, including consent withdrawal, please see our [Privacy Policy](https://track.editorialmanager.com/CL0/https://www.plos.org/privacy-policy/1/010f01958747e223-0933962d-adb4-44f3-a7e2-2bfc4cb0522a-000000/gZKKaei4NPuWZjsc1AfGEzetEFmBRed9i0q0Bowc-EA=201).

Reply: Select 'Yes' to publicly disclose identity and review history to peers.
